# Impact of Left Bundle Branch Area Pacing on Echocardiographic Parameters and Symptoms: Data From the Conduction System Pacing Italian Network Group (C‐SING) Study

**DOI:** 10.1111/jce.70355

**Published:** 2026-05-07

**Authors:** Gabriele Dell'Era, Pietro Palmisano, Daniela Dugo, Francesco Raffaele Spera, Alessandro Paoletti Perini, Gianluca Mirizzi, Luca Poggio, Luca De Mattia, Amato Santoro, Massimo Magnano, Matteo Baroni, Francesco Solimene, Davide Castagno, Donatella Ruggiero, Luca Tomasi, Antonio Rapacciuolo, Marcello Giudice, Giovanni Rovaris, Aldo Coppolino, Renzo Venanzio Iulianella, Valerio Giordano, Alessandra Tordini, Erika Taravelli, Gennaro Miracapillo, Enrico Boggio, Mario Volpicelli, Paolo Sabbatani, Francesco Pentimalli, Gianluca Manzo, Leonardo Marinaccio, Paola Napoli, Daniele Giacopelli, Giuseppe Patti

**Affiliations:** ^1^ Dipartimento Toraco‐Cardio‐Vascolare Clinica Cardiologica Novara Italy; ^2^ Cardiology Unit ‘Card. G. Panico’ Hospital Tricase Italy; ^3^ AOU Policlinico G.Rodolico ‐ San Marco Catania Italy; ^4^ Azienda Ospedaliero ‐ Universitaria Sant'Andrea Roma Italy; ^5^ Cardiology and Electrophysiology Unit, Department of Medical Specialties Azienda USL Toscana Centro, Santa Maria Nuova Hospital Florence Italy; ^6^ Fondazione Toscana “Gabriele Monasterio” Pisa Italy; ^7^ Ospedale Maggiore Di Lodi Lodi Italy; ^8^ Cardiology Department “Ca’ Foncello” Hospital Treviso Italy; ^9^ Azienda Ospedaliera Universitaria Senese Siena Italy; ^10^ Division of Cardiology, Ospedale Sant'Andrea Vercelli Italy; ^11^ Electrophysiology Unit, De Gasperis Cardio Center Niguarda Hospital Milan Italy; ^12^ Montevergine Clinic Mercogliano (AV) Italy; ^13^ Division of Cariology, Department of Medical Sciences University of Turin ‐ Città della Salute e della Scienza di Torino – Molinette Turin Italy; ^14^ Electrophysiology Unit, Cardiothoracic and vascular Department Pio XI Hospital Desio Monza and Brianza Italy; ^15^ Azienda Ospedaliera Universitaria Integrata Verona Italy; ^16^ Dipartimento di Scienze Biomediche Avanzate‐Università degli Studi di Napoli Federico II Naples Italy; ^17^ Ospedale Evangelico Betania Naples Italy; ^18^ Ospedale Umberto Parini Aosta Italy; ^19^ Cardiology Unit Fondazione IRCCS San Gerardo dei Tintori Monza Italy; ^20^ SS Annunziata Hospital Savigliano Cuneo Italy; ^21^ Ospedale Sandro Pertini Rome Italy; ^22^ Presidio Ospedaliero “San Luca” di Vallo della Lucania Salerno Italy; ^23^ Clinical and Interventional Arrhythmology, S. Maria Terni Italy; ^24^ Ospedale Santa Croce e Carle Cuneo Italy; ^25^ Cardiology Department, Electrophysiology Unit Misericordia Hospital Grosseto Italy; ^26^ Ospedale di Biella Biella Italy; ^27^ Electrophysiology Unit Ospedale Santa Maria della Pietà Nola Italy; ^28^ Ospedale “M. Bufalini” Cesena Italy; ^29^ S.S. Elettrofisiologia Cardiaca, Ospedale San Paolo Savona Italy; ^30^ Ospedale Umberto Primo, Nocera Inferiore Salerno Italy; ^31^ Department of Cardiology Immacolata Concezione Hospital Piove Di Sacco (PD) Italy; ^32^ Biotronik Italia S.P.A Cologno Monzese Italy; ^33^ University of Eastern Piedmont ‘Amedeo Avogadro’ Novara Italy

**Keywords:** cardiac pacing and clinical outcomes, conduction system pacing, left bundle branch area pacing, ventricular function

## Abstract

**Introduction:**

Left bundle branch area pacing (LBBAP) has emerged as a physiologic alternative to conventional right ventricular and biventricular pacing, yet large real‐world evidence remains limited. We aimed to assess changes in ventricular function and symptoms after LBBAP in patients with different clinical indications.

**Methods and Results:**

Consecutive patients discharged with confirmed LBBAP across 29 Italian centers underwent evaluation of echocardiographic parameters and New York Heart Association (NYHA) functional class at follow‐up.

A total of 697 patients were included: 532 with a bradycardia indication and 165 with a heart failure (HF) indication, assessed at a median follow‐up of 12.4 months. In the bradycardia group, left ventricular ejection fraction (LVEF) showed a slight improvement from 55% (interquartile range, 50–60) to 56% (52–60) (*p* = 0.027). Paced‐induced cardiomyopathy (PICM), defined as a ≥ 10% absolute LVEF reduction to < 50%, occurred in 3% of patients. Loss of LBBAP capture (*p* = 0.025) and lower LBBAP percentage (*p* = 0.024) were independent predictors of PICM.

In the HF group, LVEF improved from 35% (30–41) to 45% (36–52) (*p* < 0.001). Overall, 61.8% were classified as responders (LVEF increase ≥ 5%), rising to 73.8% among patients with ≥ 12 months of follow‐up. Higher LBBAP pacing percentage and absence of coronary artery disease independently predicted response. NYHA functional class improved significantly in both groups.

**Conclusions:**

In this large real‐world cohort, LBBAP was associated with meaningful improvements in ventricular function and symptoms in both bradycardia and HF populations. A high percentage of effective LBBAP capture is essential for optimizing outcomes, supporting its role as an effective physiologic pacing strategy.

## Graphical abstract

Left bundle branch area pacing is associated with preservation of ventricular function in patients with bradycardia indications and with significant reverse remodeling and symptomatic improvement in patients with heart failure. Maintaining a high percentage of effective LBBAP capture is critical to minimize the risk of pacing‐induced cardiomyopathy and to maximize clinical and echocardiographic benefits

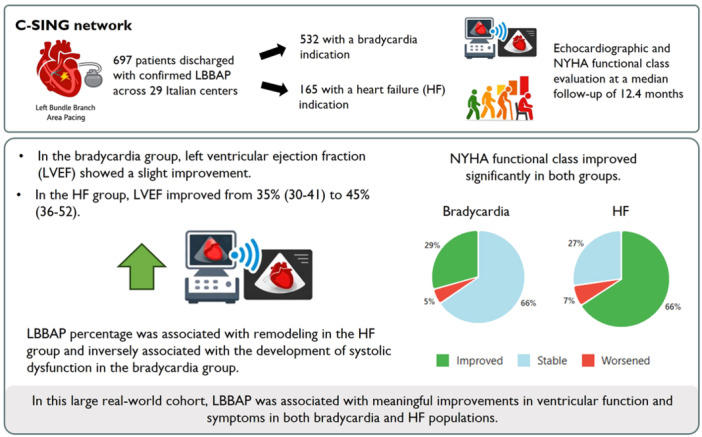

## 1 Introduction

Cardiac pacing is an evolving field of medical therapy that, over the past 60 years, has markedly improved survival and quality of life in patients with bradycardia [1, 2]. Its most recent development is conduction system pacing (CSP). By delivering permanent stimulation to the His bundle (His bundle pacing, HBP [3] or the left bundle branch area (left bundle branch area pacing, LBBAP [4], CSP directly recruits specialized His‐Purkinje fibers to maintain or restore near‐physiologic electromechanical ventricular activation. This approach preserves contractile efficiency [5, 6] and reduces pacing‐induced ventricular dysfunction [7].

LBBAP has gained widespread adoption owing to its reproducibility, technical ease, and excellent short‐ and long‐term electrical performance and safety [8, 9]. It is now recommended in several clinical scenarios [10], and growing evidence suggests benefits in heart failure outcomes and even mortality. Nevertheless, real‐world data on its performance are still limited. Most foundational studies originated from high‐volume CSP centers, and some clinicians remain skeptical about the broad implementation of LBBAP in routine practice across all hospitals.

The Conduction System Pacing Italian Network Group (C‐SING) is an Italian observational study collecting prospective clinical, procedural, and follow‐up data on CSP from centers with varying levels of expertise. The present follow‐up analysis evaluates changes in ventricular function and symptoms to assess the impact of LBBAP in patients with different pacing indications.

## 2 Methods

The C‐SING is a real‐world, multicenter, observational study designed to evaluate the practice of CSP across multiple centers in Italy. Its objectives include assessing procedural approaches, clinical indications, success rates, and outcomes in routine clinical practice. The study protocol was approved by the competent ethics committee, and all patients provided written informed consent for participation. The primary results, showing an LBBAP implantation success rate of 96.6%, have been previously published [11]. For the present analysis, which aimed to evaluate changes in echocardiographic parameters and clinical symptoms during follow‐up, the data set was locked in February 2025.

### 2.1 Study Cohort and Groups

All patients discharged after CSP implantation with effective LBBAP, available baseline (performed within 30 days before implantation) and at least one follow‐up echocardiographic examination and New York Heart Association (NYHA) functional class assessment ≥ 3 months after implantation were included. Follow‐up evaluations were performed according to routine practice at each participating center and included device interrogation with assessment of electrical parameters and a 12‑lead electrocardiogram (ECG) to assess for loss of LBBAP capture.

Patients received LBBAP based on guideline‐directed indications [2, 12], which were also used to define study groups. The bradycardia group included patients with any bradyarrhythmia indication, including atrioventricular block, sinus node disease, bradycardic atrial fibrillation, and other pacing indications in the absence of left ventricular systolic dysfunction. The heart failure group included patients implanted for heart failure‐related indications, such as standard cardiac resynchronization therapy indications, bradycardic atrial fibrillation and other conditions associated with left ventricular systolic dysfunction. The presence of left bundle branch block was defined on the 12‑lead ECG according to Strauss criteria. Paced QRS duration was measured on the 12‑lead ECG from the pacemaker stimulus artifact (spike) to the return of the last ventricular deflection to the isoelectric line.

LBBAP implantations were performed according to standard practice at each site using any commercially available delivery sheath and pacing lead (lumenless or stylet‐driven). At the end of the procedure, effective LBBAP was defined according to QRS morphology during unipolar pacing. As enrollment started in 2022, criteria initially described by Huang. et al. and later validated in subsequent studies were applied [11, 12, 13, 14, 15]. These included incomplete right bundle branch block (RBBB morphology in lead V1 and left ventricular activation time (LVAT) < 80 ms (or < 90 ms in patients with baseline bundle branch block) in lead V6. Additional criteria, considered supportive, included the presence of “fixation beats” (RBBB premature ventricular beats during lead screwing), QRS transition during threshold testing, direct recording of LBB potential, and an interpeak delay (R in V6 ‐ r′ in V1) > 33 ms. During the study, the EHRA Consensus on CSP was published [12]. Investigators were asked to classify both new and previously collected procedures according to the updated criteria. Procedures meeting the EHRA algorithm for “likely LBBP” were considered successful. Cases classified as deep septal pacing, due to the absence of a terminal R‐wave in lead V1 and no features of left conduction system pacing, were excluded from the analysis.

### 2.2 Data of Interest

Transthoracic echocardiography was performed at baseline and during follow‐up according to routine clinical practice. Investigators were asked to report the following parameters: left ventricular ejection fraction (LVEF), left ventricular end‐diastolic diameter (LVEDD), left ventricular end‐systolic diameter (LVESD), left ventricular end‐diastolic volume (LVEDV), and left ventricular end‐systolic volume (LVESV). Data on tricuspid regurgitation (TR) worsening were also collected.

In the bradycardia‐indication group, pacemaker‐induced cardiomyopathy (PICM) was defined as an absolute decrease in LVEF of ≥ 10% after device implantation, resulting in a final LVEF < 50%.

In the heart failure group, patients were classified as responders to LBBAP if follow‐up echocardiography demonstrated an absolute increase in LVEF of at least 5% relative to baseline.

NYHA functional class was assessed by investigators at baseline and follow‐up. For analysis, each patient's functional trajectory was determined by comparing follow‐up status with baseline. Patients were classified as improved if their NYHA class decreased by at least one category, stable if no change was observed, and worsened if their NYHA class increased by at least one category.

### 2.3 Statistical Analysis

Continuous variables are presented as median with interquartile range (IQR, 25th–75th percentile), and categorical variables as counts and percentages. Differences between groups were assessed using the Mann‐Whitney *U* test for continuous variables and the *χ* [2] or Fisher's exact test for binary/categorical variables.

Comparisons of echocardiographic parameters between baseline and follow‐up were performed using the Wilcoxon matched‐pairs signed‐rank test. Changes in NYHA functional class were analyzed with Bowker's test of symmetry.

Predictors of LBBAP response in the heart failure group and PICM in the bradycardia group were first assessed using univariable logistic regression models. Variables showing the strongest associations with the outcome were then entered into multivariable logistic regression models, applying the one‐in‐ten rule for events per independent variable. Results were reported as odds ratio (OR) and 95% confidence interval (CI).

A two‐sided *p*‐value < 0.05 was considered statistically significant. All analyses were performed using STATA 19.0 (StataCorp LLC, College Station, TX) and RStudio Version 2024.12.1 (Posit, PBC, Boston, MA).

## 3 Results

### 3.1 Baseline Characteristics

A total of 697 patients with confirmed effective LBBAP at hospital discharge underwent echocardiographic and NYHA functional class assessment at a median follow‐up of 12.4 months across 29 Italian centers. Baseline characteristics are summarized in Table 1. The bradycardia group included 532 patients (76.3%), while the heart failure group consisted of 165 patients (23.7%).

**Table 1:** Baseline patient characteristics.

	Total (*N* = 697)	Bradycardia indication (*n* = 532)	Heart Failure indication (*n* = 165)	*p* value
Sex, male	461 (66.3%)	339 (63.8%)	122 (74.4%)	0.012
Age (years)	78 (72–83)	79 (72–84)	77 (70–83)	0.002
NYHA functional class				< 0.001
I–II	515 (77.3%)	428 (83.9%)	87 (55.8%)	
III–IV	151 (22.7%)	82 (16.1%)	69 (44.2%)	
Medical history/Comorbidities				
Cardiomyopathy				< 0.001
None	367 (56.7%)	333 (65.6%)	34 (24.5%)	
Ischemic	130 (20.0%)	79 (15.6%)	51 (36.7%)	
Valvular	70 (10.8%)	60 (11.8%)	10 (7.2%)	
Dilated	46 (7.1%)	12 (2.4%)	34 (24.5%)	
Hypertensive	12 (1.9%)	10 (2.0%)	2 (1.4%)	
Other	22 (3.4%)	14 (2.7%)	8 (5.8%)	
Hypertension	537 (77.2%)	412 (77.4%)	125 (76.2%)	0.744
AF history	257 (37.1%)	182 (34.3%)	75 (46.3%)	0.006
Diabetes mellitus	197 (28.4%)	149 (28.1%)	48 (29.3%)	0.774
Coronary artery disease	160 (23.1%)	96 (18.1%)	64 (39.3%)	< 0.001
Renal disease	138 (19.9%)	93 (17.5%)	45 (27.4%)	0.006
BPCO	70 (10.1%)	51 (9.6%)	19 (11.7%)	0.451
Previous ictus/TIA	47 (6.8%)	31 (5.9%)	16 (9.8%)	0.083
Pre‐implant ECG				
Cardiac rhythm				0.003
Sinus rhythm	491 (71.1%)	389 (74.0%)	102 (61.8%)	
AF	181 (26.2%)	127 (24.1%)	54 (32.7%)	
Other	19 (2.7%)	10 (1.9%)	9 (5.5%)	
AV Block				
No	248 (39.6%)	153 (31.3%)	95 (69.3%)	< 0.001
I	92 (14.7%)	68 (13.9%)	24 (17.5%)	
II	125 (20.0%)	114 (23.3%)	11 (8.0%)	
III	161 (25.7%)	154 (31.5%)	7 (5.1%)	
QRS duration (ms)	122 (100–150)	110 (96–143)	150 (128–164)	< 0.001
QRS morphology				< 0.001
Normal	302 (44.9%)	271 (52.4%)	31 (20.0%)	
LBBB	189 (28.1%)	95 (18.4%)	194 (60.6%)	
RBBB and LAFB	70 (10.4%)	58 (11.2%)	12 (7.7%)	
RBBB	64 (9.5%)	61 (11.8%)	3 (1.9%)	
Nonspecific IVCD	24 (3.6%)	16 (3.1%)	8 (5.2%)	
LAFB or LPFB	23 (3.4%)	16 (3.1%)	7 (4.5%)	
Echocardiography				
Left ventricle ejection fraction (%)	55 (42–60)	55 (50–60)	35 (30–41)	< 0.001
Left ventricular end‐diastolic diameter (mm)	50 (46–55)	49 (45–52)	57 (50–62)	< 0.001
Left ventricular end‐systolic diameter (mm)	35 (30–40)	32 (29–38)	44 (38–51)	< 0.001
Left ventricular end‐diastolic volume (mL)	110 (88–143)	105 (87–126)	150 (106–188)	< 0.001
Left ventricular end‐systolic volume (mL)	51 (40–72)	46 (36–60)	93 (65‐112)	< 0.001
Main pacing indication				< 0.001
AV block	334 (48.2%)	334 (62.9%)	0 (0%)	
Sinus node dysfunction or neurocardiogenic syncope	128 (18.5%)	119 (22.4%)	9 (5.6%)	
CRT indication	99 (14.3%)	0 (0.0%)	99 (61.1%)	
AV node ablation	66 (9.5%)	42 (7.9%)	24 (14.8%)	
Atrial fibrillation with bradycardia	51 (7.4%)	36 (6.8%)	15 (9.3%)	
Non‐response to BiV CRT	15 (2.2%)	0 (0.0%)	15 (9.3%)	
Implant type				< 0.001
De‐novo	637 (91.4%)	521 (97.9%)	116 (70.3%)	
Upgrade	60 (8.6%)	11 (2.1%)	49 (29.7%)	
Device type				< 0.001
Pacemaker	564 (81.1%)	495 (93.4%)	69 (41.8%)	
CRT‐P/‐D	126 (18.1%)	35 (6.6%)	91 (55.2%)	
ICD DR	5 (0.8%)	0 (0%)	5 (3.0%)	
LBBAP lead				0.454
Stylet‐driven	378 (56.0%)	286 (55.2%)	92 (58.6%)	
Lumenless	297 (44.0%)	232 (44.8%)	65 (41.4%)	
Medication therapy at discharge				
Beta‐blockers	386 (60.4%)	242 (50.1%)	144 (92.3%)	< 0.001
Diuretics	349 (54.6%)	229 (47.5%)	120 (76.4%)	< 0.001
ACE inhibitors	234 (36.6%)	192 (39.8%)	42 (26.9%)	0.004
ARBs	131 (20.5%)	116 (24.0%)	15 (9.7%)	< 0.001
ARNI	96 (15.1%)	22 (4.6%)	74 (47.4%)	< 0.001
Digitalis	60 (9.4%)	36 (7.5%)	24 (15.5%)	0.003
Anticoagulants	261 (40.9%)	178 (36.9%)	83 (53.5%)	< 0.001
Antiplatelet	221 (34.5%)	165 (34.0%)	56 (35.9%)	0.668
Antiarrhythmics	66 (10.4%)	49 (10.3%)	17 (11.0%)	0.800

*Note:* Data are shown as median (interquartile range) and *n* (% calculated on non‐ missing data) for categorical variables.

Abbreviations: ACE, angiotensin converting enzyme; ARBs, angiotensin receptor blockers; ARNI, angiotensin receptor‐neprilysin; AF, atrial fibrillation; AV, atrio‐ventricular; COPD, chronic obstructive pulmonary disease; CRT‐P/‐D, cardiac resynchronization therapy pacemaker/defibrillator; ECG, electrocardiogram; ICD DR, dual‐chamber implantable cardioverter defibrillator; IVCD, intraventricular conduction delay; LBBB, left bundle branch block; LVEF, left ventricle ejection fraction, NYHA, New York Heart Association; RBBB, right bundle branch block; TIA, transient ischemic attack.

As expected, baseline characteristics differed significantly between the two groups (Table 1). In the bradycardia group, 63.8% of patients were male, and the median age was 79 years (72–84). Atrioventricular block was the predominant pacing indication (62.9%), nearly all procedures were de‐novo implantations (97.9%), and the median LVEF was 55% (50%–60%). Additionally, 55 patients (11.1%) had preserved LVEF (≥ 50%) but presented with an advanced NYHA functional class.

In the heart failure group, 74.4% of patients were male, and the median age was 77 years (70–83). Approximately half (44.2%) presented with NYHA functional class III–IV, and the median LVEF was 35% (30%–41%). A complete left bundle branch block was present in 60.6% of patients, with a median intrinsic QRS duration of 150 ms (128–164). About one‐third (29.7%) of the procedures in this group consisted in system upgrades from a prior conventional device.

### 3.2 LBBAP: Acute and Follow‐up Details

All procedures were classified as successful, with confirmation of effective LBBAP at the time of hospital discharge using either stylet‐driven (56.0%) or lumenless (44.0%) leads (Supporting Information S1: Table S1). Left fascicular pacing was the most frequently reported capture type (69.1%), followed by left bundle branch pacing (25.0%) and left ventricular septal pacing (5.9%). Overall, the median paced QRS duration was 120 ms (110–130), LVAT was 71 ms (65–79), and the V6–V1 interpeak interval was 42 ms (35–50). The sensing amplitude, pacing threshold at 0.4 ms, and impedance for the LBBAP lead were 9.0 mV (6.3–12.2), 0.75 V (0.5–1.1), and 630 Ohm (505–747), respectively. None of the LBBAP electrical parameters differed significantly between groups (Supporting Information S1: Table S2).

During a median follow‐up of 12.4 months (4.6–23.7), six patients (0.9%) required revision due to LBBAP lead macro‐dislodgement: five patients (0.9%) in the bradycardia group and one (0.6%) in the heart failure group.

In addition, loss of LBBAP capture at follow‐up was reported in 16 patients (2.3%), including 12 patients (2.2%) in the bradycardia group and 4 patients (2.4%) in the heart failure group. In all these cases, ventricular pacing remained effective, but without evidence of left conduction system or left ventricular septal capture.

The median percentage of LBBAP at follow‐up was 99% (86%–100%).

### 3.3 Echocardiographic Measurements

**Figure 1:**
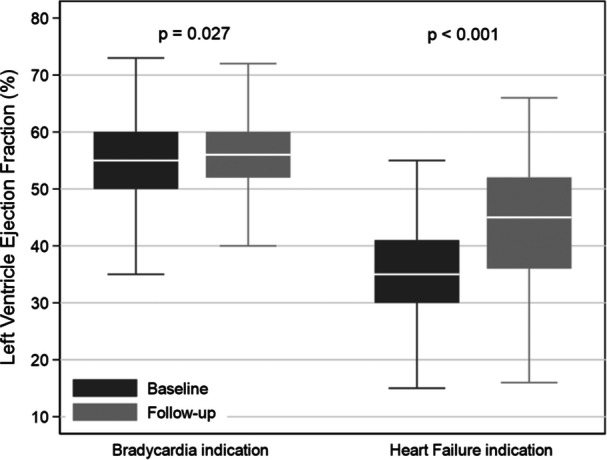
Boxplots showing left ventricular ejection fraction at baseline and at follow‐up in the bradycardia and heart failure groups.

Figure 1 displays LVEF at baseline and follow‐up. In the bradycardia group, a small but statistically significant improvement was observed, from 55% (50–60) to 56% (52–60) (*p* = 0.027). The improvement was more pronounced in the heart failure group, where LVEF increased from 35% (30–41) to 45% (36–52) (*p* < 0.001).

**Figure 2:**
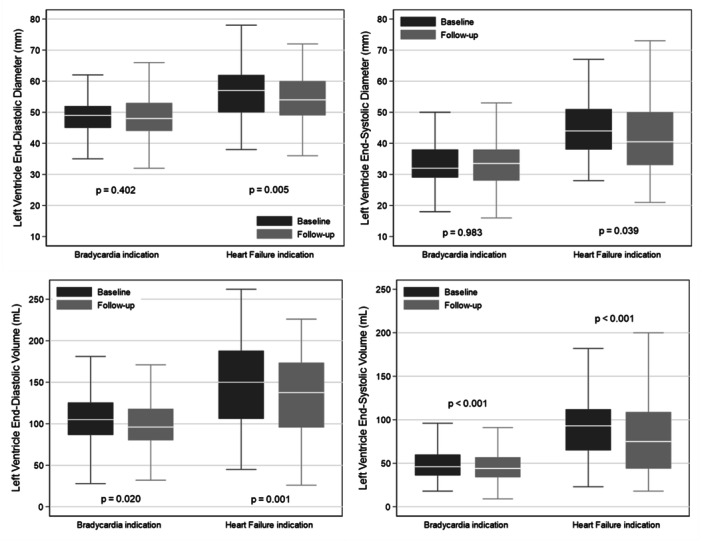
Boxplots showing left ventricular end‐diastolic and end‐systolic diameters, as well as end‐diastolic and end‐systolic volumes, at baseline and follow‐up in the bradycardia and heart failure groups.

Similar trends were observed for other echocardiographic parameters (Figure 2). In the bradycardia group, LVEDD and LVESD remained stable between baseline and follow‐up (*p* = 0.402 and *p* = 0.983, respectively), while slight but statistically significant reductions were observed for LVEDV (from 105 mL [87–126] to 96 mL [80–118], *p* = 0.020) and LVESV (from 46 mL [36–60] to 44 mL [34–57], *p* < 0.001).

In the heart failure group, marked reductions were observed for LVEDD (*p* = 0.005) and LVESD (*p* = 0.039), from baseline values of 57 mm (50–62) and 44 mm (38–51) to follow‐up values of 54 mm (49–60) and 40 mm (33–50), respectively. Likewise, LVEDV and LVESV significantly decreased (*p* = 0.001 and *p* < 0.001) from 150 mL (106–188) and 93 mL (65–112) to 137 mL (95–173) and 75 mL (44–109), respectively.

At follow‐up, TR assessment was available for 302 patients. Compared with the pre‐implant evaluation, 231 patients (76.5%) showed stable TR grading, 47 (15.6%) demonstrated improvement, and 24 (7.9%) exhibited worsening. Among those with worsening TR, 16 patients progressed from mild to moderate TR, one from mild to severe, two from moderate to severe, two from moderate to massive, and three from severe to massive. The proportion of patients with improved TR was lower in the bradycardia group than in the heart failure group (*n* =  32, 13.5% vs. *n* =  15, 23.4%; *p* = 0.050). No significant differences were observed between groups in the prevalence of TR worsening (*n* =  16, 6.7% vs. *n* =  8, 12.5%; *p* = 0.129). Neither lead type (stylet driven vs. lumenless) nor the type of LBB capture (conduction system capture subtype) showed any association with TR improvement or worsening (all *p* > 0.30).

### 3.4 Predictors of PICM and LBBAP Response

Among the 532 patients with a bradycardia indication, 16 (3.0%) developed PICM, defined as an absolute LVEF reduction ≥ 10% to a final LVEF < 50%. At univariable analysis, coronary artery disease, loss of LBBAP capture during follow‐up, and LBBAP percentage were significantly associated with PICM risk (Table 2). At multivariable analysis, loss of LBBAP capture remained associated with a markedly increased risk of PICM (OR 28.1, 95%CI 6.72–117; *p* = 0.025), while each 1% increase in LBBAP percentage was associated with a 2% reduction in PICM risk (OR 0.98, 95%CI 0.97–0.99; *p* = 0.024).

**Table 2:** Predictors of paced‐induced cardiomyopathy in patients with bradycardia indication.

Independent variable	Univariable analysis		Multivariable analysis	
	OR (95% CI)	*p* value	OR (95% CI)	*p* value
Female	0.58 (0.18–1.82)	0.351		
Age (years)	1.03 (0.97–1.09)	0.335		
Coronary artery disease	2.83 (1.00–7.99)	0.049		
AF at implantation	1.88 (0.67–5.28)	0.231		
LBBB	1.22 (0.65–2.30)	0.541		
Baseline LVEF	1.03 (0.96–1.11)	0.353		
De‐novo implantation	0.30 (0.04–2.47)	0.261		
Loss of LBBAP capture during follow‐up	33.0 (9.01–120)	< 0.001	28.1 (6.72–117)	< 0.001
Percentage of LBBAP (%)	0.98 (0.97–0.99)	0.002	0.98 (0.97–0.99)	0.024
LVAT	1.03 (0.98–1.07)	0.208		
Paced QRS duration (ms)	1.00 (0.97–1.03)	0.934		

Abbreviations: AF, atrial fibrillation; CI, confidence interval; LBBAP, left bundle branch area pacing; LVAT, left ventricular activation time; LVEF, left ventricle ejection fraction; OR, odds ratio.

Among the 165 patients with a heart failure indication, 102 (61.8%) were classified as responders, defined as an absolute LVEF improvement of ≥ 5%. The responder rate increased to 70.8% (85/120) in patients with ≥ 6 months of LBBAP and to 73.8% (62/84) in those with ≥ 12 months of follow‐up. At univariable analysis, female sex, coronary artery disease, baseline LVEF ≤ 35%, LBBAP percentage, LVAT, and paced QRS duration were significantly associated with echocardiographic response (Table 3). At multivariable analysis, only the absence of coronary artery disease (OR 0.30, 95%CI 0.11–0.86; *p* = 0.025) and the percentage of LBBAP (OR 1.03, 95%CI 1.00–1.05; *p* = 0.023) remained independent predictors of response. Of note, 26.1% of patients (43/165) demonstrated a super‑response to LBBAP, defined as an absolute LVEF improvement of ≥ 20% or an increase in LVEF to > 50%.

**Table 3:** Predictors of LBBAP response in patients with heart failure indication.

Independent variable	Univariable analysis		Multivariable analysis	
	OR (95% CI)	*p* value	OR (95% CI)	*p* value
Female	2.82 (1.24–6.39)	0.013	1.86 (0.52–6.65)	0.338
Age (years)	1.03 (0.99–1.06)	0.139		
Coronary artery disease	0.41 (0.21–0.78)	0.007	0.30 (0.11–0.86)	0.025
AF at implantation	1.05 (0.53–2.08)	0.883		
LBBB	1.21 (0.65–2.96)	0.541		
Baseline LVEF ≤ 35%	2.02 (1.06–3.82)	0.032	1.77 (0.65–4.79)	0.258
De‐novo implantation	1.49 (0.75–2.94)	0.250		
Loss of LBBAP capture during follow‐up	0.61 (0.08–4.44)	0.626		
Percentage of LBBAP at follow‐up (%)	1.02 (1.00–1.03)	0.020	1.03 (1.00–1.05)	0.023
LVAT	0.97 (0.95–0.99)	0.018	0.98 (0.94–1.01)	0.240
Paced QRS duration (ms)	0.98 (0.96–0.99)	0.016	1.00 (0.97–1.03)	0.944

Abbreviations: AF, atrial fibrillation; CI, confidence interval; LBBAP, left bundle branch area pacing; LVAT, left ventricular activation time; LVEF, left ventricle ejection fraction; OR, odds ratio.

### 3.5 NYHA Functional Class Evaluations

**Figure 3:**
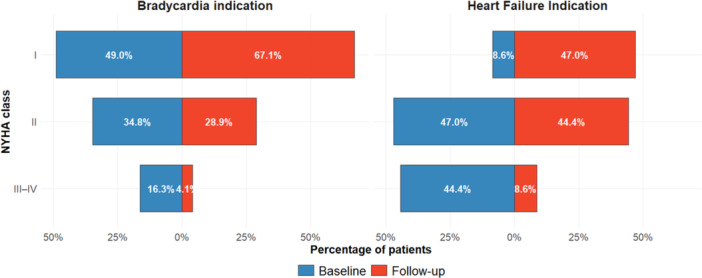
Changes in NYHA functional class from baseline to follow‐up in the two study groups.

**Figure 4:**
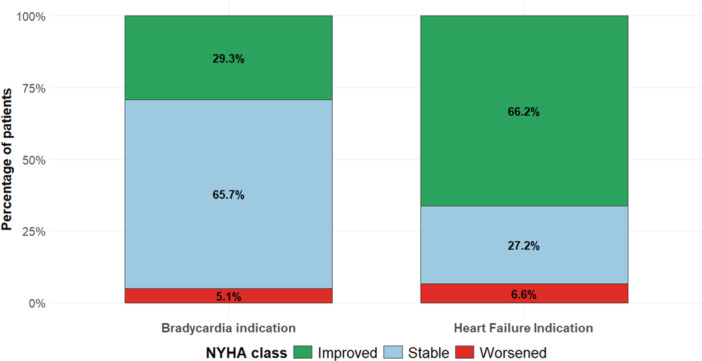
Distribution of NYHA functional class changes (improved, stable, worsened) at follow‐up among patients in the bradycardia and heart failure groups.

Significant improvements in NYHA functional class were observed in both groups (Figure 3). In the bradycardia group, the prevalence of NYHA class I increased from 49.0% to 67.1%, while class III–IV decreased from 16.3% to 4.1% (*p* < 0.001). The proportions of patients with improved, stable, and worsened NYHA class were 29.3%, 65.7%, and 5.1%, respectively (Figure 4). A significant functional improvement was also seen in the subgroup of patients with preserved LVEF but severe heart failure symptoms at baseline (Supporting Information S1: Table S3).

In the heart failure group, the prevalence of NYHA class I increased from 8.6% to 47.0%, while class III–IV decreased from 44.4% to 8.6% (*p* < 0.001). The proportions of patients with improved, stable, and worsened NYHA class were 66.2%, 27.2%, and 6.6%, respectively (Figure 4).

## 4 Discussion

In this large, real‐world multicenter cohort, LBBAP was associated with favorable echocardiographic and clinical trajectories across a broad spectrum of pacing and heart failure indications. Among patients implanted for bradyarrhythmias, ventricular function remained largely stable, and the incidence of PICM was low, particularly when high LBBAP pacing percentages were maintained. In the heart failure population, LBBAP resulted in significant reverse remodeling and meaningful improvements in LVEF. Functional status also improved in both groups, most prominently in patients with baseline heart failure, reinforcing the clinical value of physiologic pacing in routine practice. The safety profile of LBBAP was confirmed by the very low rate of lead macro‐dislodgement requiring revision (≈1%) over a median follow‐up of approximately 1 year. To our knowledge, this study represents the first report on the evolution of ventricular function after LBBAP within a large, collaborative, real‐world network, encompassing centers with heterogeneous experience but a shared commitment to the technique and peer‐to‐peer support.

Evidence supporting the beneficial effects of LBBAP on ventricular function is steadily accumulating [13, 14, 15]. Reports demonstrating stable long‐term electrical performance [8, 11], the effectiveness of automated follow‐up and programming algorithms [16, 17], and reassuring procedural safety [18, 19] further strengthen the rationale for its use. Nevertheless, ongoing evaluation in large, real‐world settings remains essential, as the technique continues to evolve, new tools become available, and an increasing number of clinicians adopt LBBAP in daily practice.

In patients with bradycardia indications, we observed a low (3%) incidence of ventricular dysfunction development, which was strongly associated with loss of LBBAP capture and lower pacing percentages. Only limited data exist on this topic, and a recent single‐center study reported a higher PICM incidence (7.5% at 26 weeks), largely attributable to the absence of true LBBAP (deep septal pacing) [20]. Taken together, these findings suggest that in patients with preserved LVEF, operators should make a concerted effort to achieve true LBBAP and to monitor for potential loss of capture during follow‐up. Under these conditions, LBBAP has the potential to serve as a first‐line physiological pacing strategy for preventing PICM, a concept supported by the recent MELOS‐RELOADED study, which reported long‐term survival benefits compared with conventional myocardial pacing [21].

As expected, the greatest echocardiographic improvements were observed in patients with baseline ventricular dysfunction. Significant reductions in LV diameters and volumes indicated favorable reverse remodeling, analogous to what has long been documented with BivP [22]. In our heterogeneous heart failure population, characterized by a 61% prevalence of complete left bundle branch block and notable proportions of device upgrades (30%) and prior non‐responders to CRT (9%), the overall response rate to LBBAP was 62%, increasing to 71% with ≥ 6 months of follow‐up and to 74% with ≥ 12 months. These results align with those of conventional CRT and with the growing recognition that reverse remodeling may continue to progress beyond the first 6 months [23]. As with BivP, a higher percentage of LBBAP capturing and the absence of coronary artery disease were independently associated with a favorable response.

NYHA functional class improved consistently with the echocardiographic findings, with the most pronounced benefit observed in patients with baseline heart failure and impaired functional status. This trend was also evident in the subgroup of patients with heart failure and preserved LVEF (Supporting Information S1: Table S3), where the combination of physiological pacing and backup heart rate support may have further contributed to an improvement in functional capacity and quality of life [24]. Progression of TR was limited, with only a small proportion of patients developing severe regurgitation. Concerns regarding tricuspid interference arose during the early experience with HBP, where septal leaflet impingement and subvalvular interaction were considered non‐negligible risks [25]. LBBAP appears less problematic in this regard: a more distal lead position reduces the likelihood of entanglement or leaflet perforation, and the proximal slack often lies in an intercommissural position rather than in the conventional apical trajectory [26]. Our observations support this hypothesis by showing minimal lead‐related TR progression.

Mechanistic studies have previously highlighted the beneficial effects of LBBAP on myocardial synchrony and energetic efficiency [8, 9, 27]. Our findings suggest that these physiological improvements translate into better preservation or enhancement of LVEF and may, over the long term, reduce the burden of heart failure related to PICM or untreated ventricular dysfunction [17, 18, 25, 28, 29].

We therefore believe that these results, together with the growing evidence base and the anticipated findings of ongoing randomized trials, should encourage the broader adoption of LBBAP as an essential tool in contemporary practice. Establishing structured peer‐to‐peer networks, multicenter registries, and standardized training pathways will be crucial for supporting clinicians and ensuring consistent, high‐quality implementation of this promising pacing strategy.

## 5 Study Limitations

This case‐only observational study lacked a control arm for comparing LBBAP with alternative pacing strategies, such as BivP or conventional myocardial pacing. However, because participating clinicians followed current guideline recommendations [2, 10, 22], the heart failure group consisted of patients for whom LBBAP was selected over conventional epicardial stimulation via a coronary sinus lead. This context should be considered when interpreting our findings, as these patients have only recently gained access to a satisfactory alternative treatment. In the bradycardia cohort, the detrimental effects of conventional myocardial pacing in patients receiving more than 20% ventricular pacing are well established [7], and given that our patients exhibited very high ventricular pacing percentages (median 99%), the observed outcomes remain particularly noteworthy. We were unable to evaluate the potential impact of atrial pacing rate in specific subgroups [24], as detailed pacemaker programming parameters were not systematically collected in the registry. Furthermore, although baseline medical therapy was recorded, subsequent changes in drug treatment during follow‑up were not consistently tracked.

Another limitation is the absence of predefined follow‐up time points and the lack of a centralized core laboratory for electrocardiology and echocardiographic analysis. These constraints reflect the observational design, which did not interfere with routine clinical practice. In many participating hospitals, echocardiographic follow‐up is not routinely performed in clinically stable or asymptomatic patients but is reserved for those who develop heart failure or functional deterioration. Although this cannot be directly confirmed from our data set, maintenance or improvement of LVEF appears especially relevant in this selected population.

Finally, the median follow‐up duration of approximately 1 year, while sufficient to evaluate the clinical and echocardiographic effects of LBBAP in patients with heart failure, as pivotal BivP trials used similar or shorter timeframes, may underestimate the long‐term incidence of PICM. Nevertheless, because LVEF reduction was rare and ventricular pacing percentages remained high, we believe the study provides a reasonable estimate of PICM risk within the examined period.

## 6 Conclusions

In this large, real‐world multicenter cohort, LBBAP demonstrated favorable safety, together with meaningful clinical and echocardiographic benefits across both bradycardia and heart failure populations. Ventricular function was largely preserved in patients paced for bradyarrhythmias, with a low incidence of PICM that was strongly linked to loss of LBBAP capture. Among patients with heart failure, LBBAP promoted significant reverse remodeling, substantial improvement in LVEF, and marked gains in functional status. High percentages of LBBAP capture consistently predicted better outcomes in both groups. These findings support the role of LBBAP as an effective and physiologic pacing strategy in routine practice across different indications.

## Disclosure

Gabriele Dell'Era received fees for lectures and proctorship on conduction system pacing by Abbott, Biotronik and Boston Scientific; Davide Castagno received fees for lectures and participation in advisory boards for Biotronik and Boston Scientific and his institution has received research support from Boston Scientific outside the submitted work. Paola Napoli and Daniele Giacopelli are employees of Biotronik. No other conflicts of interest to disclose.

## Funding

The authors have nothing to report.

## Supporting information

Supporting File

## Data Availability

The data that support the findings of this study are available from the corresponding author upon reasonable request.
