# Supplementary material for: Impact of Left Bundle Branch Area Pacing on Echocardiographic Parameters and Symptoms: Data From the Conduction System Pacing Italian Network Group (C‐SING) Study
Source: J Cardiovasc Electrophysiol. 2026 May 7;37(7):1432–41. doi: 10.1111/jce.70355 (PMC13372409; doi:10.1111/jce.70355)
Supplement: Supplementary file 1 — Supporting File [file JCE-37-1432-s001.docx]

Supplementary Material

**Impact of Left Bundle Branch Area Pacing on Echocardiographic Parameters and Symptoms: Data from the Conduction System pacing Italian Network Group (C-SING) study**

Gabriele Dell’Era, et al.

**Table S1**. Left bundle branch area pacing (LBBAP) lead type

|  | **Total**  **(N=697)** |
| --- | --- |
| Stylet-driven | 378 (56.0%) |
| Solia S 60, Biotronik | 290 (43.0%) |
| Ingevity +, Bostron Scientific | 63 (9.3%) |
| Tendril STS, Abbott | 21 (3.1%) |
| Vega R58, Microport | 4 (0.6%) |
| Lumenless | 297 (44.0%) |
| Select Secure 3830, Medtronic | 297 (44.0%) |

Data are shown as n (% calculated on non- missing data).

**Table S2**. Characteristics of LBBAP at implantation

|  | **Total**  **(N=697)** | **Bradycardia indication**  **(n=532)** | **Heart Failure indication**  **(n=165)** | **P value** |
| --- | --- | --- | --- | --- |
| LBBAP capture type |  |  |  | 0.174 |
| LFP | 304 (69.1%) | 242 (71.8%) | 62 (60.2%) |  |
| LBBP | 110 (25.0%) | 77 (22.8%) | 33 (32.0%) |  |
| LVSP | 26 (5.9%) | 18 (5.3%) | 8 (7.7%) |  |
| *Post-implant ECG* |  |  |  |  |
| Paced QRS duration (ms) | 120 (110-130) | 120 (109-130) | 120 (110-130) | 0.458 |
| LVAT (ms) | 71 (65-79) | 71 (65-78) | 72 (65-80) | 0.081 |
| V6–V1 interpeak interval (ms) | 42 (35-50) | 42 (34-50) | 42 (35-50) | 0.880 |
| *LBBAP electrical parameters* |  |  |  |  |
| R-wave amplitude | 9.0 (6.3-12.2) | 9.0 (6.7-12.3) | 9.0 (6.0-12.0) | 0.560 |
| Pacing threshold (V) @0.4 ms | 0.75 (0.50-1.1) | 0.70 (0.50-1.0) | 0.80 (0.50-1.4) | 0.043 |
| Pacing impedance (Ohm) | 630 (505-747) | 631 (507-746) | 605 (480-750) | 0.216 |

Data are shown as median (interquartile range) and n (% calculated on non-missing data) for categorical variables.

The LBBAP capture type was determined by the implanting investigator using all available intraprocedural information—paced 12‑lead ECGs recorded at different sweep speeds and fluoroscopic imaging—according to the recommendations of the EHRA clinical consensus statement on conduction system pacing implantation.

Abbreviations: ECG, electrocardiogram; LBBAP, left bundle branch area pacing; LBBP, left bundle branch pacing; LFP, left fascicular pacing; LVAT, left ventricle activation time; LVSP, left ventricular septal pacing.

**Table S3**. Functional improvement in the subgroup of patients with preserved LVEF but severe heart failure symptoms at baseline

|  | NYHA functional class at follow-up | | |
| --- | --- | --- | --- |
|  | I | II | III |
| Patients with bradycardia indication and HFpEF (n=59) | 23 (40%) | 25 (43%) | 10 (17%) |

HFpEF, heart failure with preserved ejection fraction.
